# Polyphenols with Antiulcerogenic Action from Aqueous Decoction of Mango Leaves (*Mangifera indica* L.)

**DOI:** 10.3390/molecules14031098

**Published:** 2009-03-10

**Authors:** Juliana Aparecida Severi, Zeila Pinheiro Lima, Hélio Kushima, Alba Regina Monteiro Souza Brito, Lourdes Campaner dos Santos, Wagner Vilegas, Clélia Akiko Hiruma-Lima

**Affiliations:** 1 Pharmacos and Drugs Department, Pharmaceutical Sciences Faculty, São Paulo State University-UNESP, c.p.355, Zip Code: 14801-902, UNESP, Araraquara, SP, Brazil; E-mail: juseveri@yahoo.com.br (J-A.S.); 2 Physiology Department, Biosciences Institute, São Paulo State University-UNESP, c.p. 510, Zip Code: 18618-000, Botucatu, SP, Brazil; E-mail: zeilabio@hotmail.com (Z-P.L.), heliokushima@gmail.com (H.K.); 3 Physiology and Biophysics Department, Biology Institute, Campinas State University-UNICAMP, c.p. 6109, Zip Code: 13083-970, Campinas, SP, Brazil; E-mail: abrito@unicamp.br (A-R.M.); 4 Organic Chemistry Department, Chemistry Institute, São Paulo State University-UNESP, c.p. 355, Zip Code: 14800-900, UNESP, Araraquara, SP, Brazil; E-mail: vilegasw@gmail.com (W.V.), loursant@iq.unesp.br (L-C.S.)

**Keywords:** *Mangifera indica*, Antiulcer gastric, Mangiferin, Benzophenone, Phenolic compounds.

## Abstract

This study was designed to determine the gastroprotective effect of a *Mangifera indica* leaf decoction (AD), on different experimental models in rodents. The administration of AD up to a dose of 5 g/kg (p.o.) did not produce any signs or symptoms of toxicity in the treated animals, while significantly decreasing the severity of gastric damage induced by several gastroprotective models. Oral pre-treatment with AD (250, 500 or 1000 mg/kg) in mice and rats with gastric lesions induced by HCl/ethanol, absolute ethanol, non-steroidal anti-inflammatory drug (NSAID) or stress-induced gastric lesions resulted in a significant decrease of said lesions. Phytochemical analyses of AD composition demonstrated the presence of bioactive phenolic compounds that represent 57.3% of total phenolic content in this extract. Two main phenolic compounds were isolated, specifically mangiferin (C-glucopyranoside of 1,3,6,7-tetrahydroxyxanthone) and C-glucosyl-benzophenone (3-C-β-D-glucopyranosyl-4’,2,4,6-tetrahydroxybenzophenone). These findings indicate the potential gastroprotective properties of aqueous decoction from *M. indica* leaves.

## Introduction

Acid related disorders are common conditions in the general population. Furthermore, clinical experience has shown that acid-related symptoms as well as duodenal ulcers return quickly after cessation of drug treatments, raising the possibility of rebound hypersecretion [1]. Therapy in the future will continue to focus on control of acid secretion and subsequent reversal of mucosal damage and inflammation [2]. 

*Mangifera indica* L. (Anacardiaceae) is one of the most important tropical plants marketed in the world [3]. It is a large tree that grows in tropical and subtropical regions, whose fruits are widely appreciated by the population. There are many traditional medicinal uses for the bark, roots and leaves of *M. indica* throughout the globe [3]. This plant was listed in TRAMIL (Program of Applied Research to Popular Medicine in the Caribbean) as an agent for the treatment of diarrhea, fever, gastritis and ulcers [4, 5]. Phytochemical research from different parts of *M. indica* has demonstrated the presence of phenolic constituents, triterpenes, flavonoids, phytosterols, and polyphenols [6,7,8,9,10]. This species is purported to posses numerous therapeutic uses including analgesic, anti-inflammatory [11], immunostimulant [12,13,14], antioxidant [15,16,17], spasmolytic, antidiarrhea [18], dyslipidemic [19], antidiabetic [20,21], antiamebic [22], anthelminthic, antiallergic [23] and antibacterial applications [24]. Moreover, a previous work carried out by our group showed antiulcerogenic and healing effects of aqueous decoction of *M. indica* flowers [25]. However, the great demand for this decoction has diminished the availability of *M. indica* fruits that are widely appreciated by the population as food. Therefore, the study aimed to evaluate the antiulcer activity of the aqueous decoction (AD) obtained from the leaves of *Mangifera indica* using different *in vivo* experimental models in rodents followed by its phytochemical investigation.

## Results and Discussion

The gastroprotective action exerted by an aqueous decoction (AD) of *Mangifera indica* leaves emerged from an ethnobotanical inventory from the Traditional Medicine of the Islands (TRAMIL project) for development of the Caribbean Medicinal Plant Pharmacopoeia. As part of this pharmacological study, the AD obtained from leaves of *M. indica* was first investigated in relation to its acute toxicity *in vivo*. This application suggests that there would be no toxic effects from the administration of this decoction. A single oral administration of AD from *M. indica* via the oral route at dose of 5 mg/Kg did not produce any signs or symptoms of toxicity in the treated animals. During the following 14 days after the administration of AD, no animals died and there were no significant alteration in water or food consumption. At autopsy, no significant change or lesion was observed in the viscera of any animal (Table 1). Loomis and Hayes [26] described the classification of some chemical agents into categories of toxicity in which the dose of 5 g/Kg was categorized as practically nontoxic, a result which indicates that this preparation from *M*. *indica* has no acute toxicological effect when administered orally. These results demonstrated the need for continuation of pharmacological studies on the oral administration of this decoction and motivated us to continue with the assays.

**Table 1:** Evaluation of the acute toxicity of aqueous decoction (AD) of *M. indica* leaves (5 g/Kg, p.o.) in male Swiss mice (n=10).

Weight (g)	Control	AD
Corporal	43.00 ± 0.63	43.00 ± 1.18
Kidney	0.59 ± 0.31	0.52 ± 0.02
Liver	2.00 ± 0.72	1.82 ± 0.09
Heart	0,23 ± 0.01	0.18 ± 0.02
Lungs	0.26 ± 0.02	0.23 ± 0.01
Mortality	0/10	0/10

Results are mean ± S.E.M; n=10. Student’s t test.

Not significant p>0.05.

**Figure 1:**
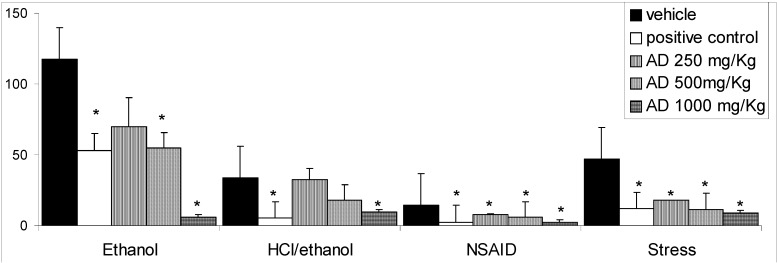
Effects of different doses of aqueous decoction (AD) of *Mangifera indica* leaves on models of gastric lesions induced in mice and rats (mm).

Intragastric application of absolute ethanol has long been used as a reproducible method to induce gastric lesions in experimental animals [27]. Recent studies have demonstrated that ROS (reactive oxygen species) such as superoxide anion radical, hydroxyl radical (OH) and lipid peroxidation play an important role in pathogenesis of acute gastric damage induced by ethanol [28]. ROS provoke severe changes at the cellular level leading to cell death because of their extreme reactivity [29]. The oral administration of AD before the induction of gastric lesion by absolute ethanol significantly decreased the gastric lesions relative to the control group in which 500 mg/Kg generated 55% of inhibition, while 1,000 mg/Kg produced 95% of inhibition (Figure 1). The gastric injury caused by ethanol plus hydrochloric acid also can be seen in Figure 1. The pretreatment with AD (at doses of 500 or 1,000 mg/Kg) before HCl/ethanol solution significantly (p < 0.05) decreased the severity of lesions by 47 and 70%, respectively. These agents confer a direct topical effect on gastric mucosa, which is an undesirable *sequela* and a suitable model to investigate products with possible cytoprotective activity [30]. When the gastric mucosal defense is compromised by exogenous agents such as ethanol, these compounds penetrate into gastric mucosa and damage the mucosal microvessels [31]. The AD was able to reduce stress-induced ulcers diminishing the lesions in relation to the control group at all AD doses (250, 500 and 1,000 mg/Kg). Stress causes sympathetic stimulation of the stomach which induces direct arteriolar vasoconstriction and gradually reduces the blood flow to the stomach, leading to local hypoxia and ischemia [32]. Ischemia increases the leakage of O_2_ from the mitochondrial electron transport chain [33] and facilitates the availability of “redox-active” copper and iron. This leakage of O_2_ can cause lipid peroxidation and lead to a loss of membrane fluidity, impairment of both ion transport and membrane integrity, and a loss of cellular functions [34]. These two factors play a joint role in gastric ulcers induced by stress. 

Results are mean ± S.E.M.: Ethanol: F_(4;20)_= 7.05, HCl/ethanol: F_(4; 27)_ = 6.95;

NSAID: F_(4; 33)_ = 7.23 and Stress: F_(4;28)_ = 7.54. Dunnett’s test * p<0.05.

NSAIDs are the most widely used pharmacological agents in the treatment of pain, inflammation and fever. However 15-30% of patients taking NSAIDs develop peptic ulcers [35]. It is well known that prostaglandins (PGs) play a key role in protecting the gastric mucosa against injury caused by a variety of necrotizing agents [36]. Under the NSAID model, AD showed significant (p < 0.05) activity at doses of 250 (61%) and 500 mg/Kg (75%), 1,000 mg/Kg (82%) in relation to vehicle-treated animals. This drop in the severity of lesions by augmentation of AD doses demonstrated an important difference between aqueous decoction of *M. indica* obtained from flowers and leaves. Lima *et al*. [25] concluded that the absence of gastroprotective activity at the highest dose of AD obtained from flowers probably was due to the chemical compounds in this preparation which induced modulation of endogenous prostaglandins. 

**Figure 2:**
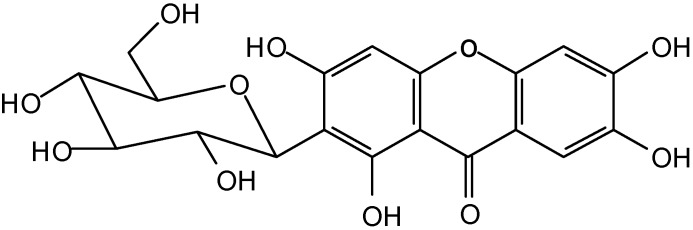
Structure of Mi1, mangiferin, isolated from *M. indica* leaves.

**Figure 3:**
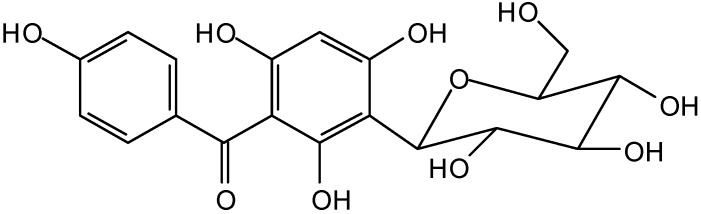
Structure of Mi2, benzophenone *C*-glycoside, isolated from *M. indica* leaves.

The aqueous decoction from *Mangifera indica* leaves presents a different phytochemical profile from the flowers. The phytochemical study of AD of leaves leads to isolation of two phenolic compounds, Mi1 and Mi2. Compound Mi1 was obtained as an amorphous yellow solid. The ^13^C-NMR spectrum of compound Mi1 was in complete agreement with the one reported for mangiferin (Figure 2), a *C*-glucopyranoside of 1,3,6,7-tetrahydroxyxanthone [37], occurring in a variety of plants. The compound Mi2 was identified by spectral analysis as 3-*C*-β-D-glucopyranosyl-4’,2,4,6-tetrahydroxybenzophenone (Figure 3), a *C*-glucosylbenzophenone.

**Figure 4:**
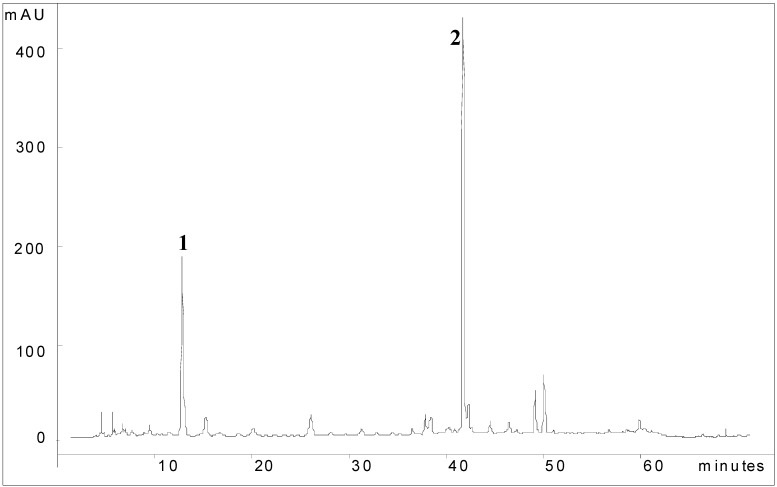
HPLC-UV-PDA fingerprint of the AD of *Mangifera indica* leaves 1: benzophenone glycoside; 2: mangiferin. Detection at 254 nm. Chromatographic conditions: see text.

The HPLC-PDA chromatogram of AD showed the presence of two intense peaks at 254 nm with retention times of 11.6 and 40.5 min, respectively (Figure 4). The UV spectra of these peaks, together with the analysis of retention time of standard compounds, indicated the isolated Mi2 and Mi1, respectively. The total phenolic content obtained in AD from leaves was greater than the percentage (57.3%) present in AD of *M. indica* flowers [25].

Many experiments have also demonstrated the role of antioxidants in preventing various human diseases by preventing oxidative stress and damage in biological tissues [38]. Due to the presence of conjugated ring structures and hydroxyl groups, most phenolic compounds have the potential to function as antioxidants by scavenging the superoxide anion, hydroxyl radical and peroxy radical or quenching singlet oxygen, thus inhibiting lipid peroxidation in biological systems. *M. indica* represents a rich source of phenolic compounds [39,40].

Mangiferin (2-*β*-D-glucopyranosyl-1,3,6,7-tetrahydroxy-9H-xanthen-9-one), is a xanthone commonly found in various parts of *M. indica* and has attracted considerable interest in view of its numerous therapeutic effects including, for example, antitumor [41], antiviral [42], antidiabetic [43], anti-inflammatory [44] and potent antioxidant [17,45,46] properties. A close association between the antioxidant activity and antiulcerogenic effect has also been also reported in many studies [38]. One recent work described the antiulcerogenic activity of mangiferin against gastric damage induced by ethanol and indomethacin [47]. This polyphenol is capable of acting as an antioxidant through many mechanisms available *in vitro*: primarily as potent scavenger of free radicals [48]; as modulating the activation and functionality of rat macrophages, through partial inhibition of ROS and RNS production [13] and by its ability to bind to iron-complex ions (Fe^2+^/^3+^), resulting in membrane lipid peroxidation protection [49]. The catechol moiety with a 6,7-dihydroxylated structure, together with its aromatic bonds, has been proposed as being responsible for its antioxidant property [45]. During protection afforded against free radical production, the catechols are oxidized, generating products including semiquinone radicals and quinones.

Another important class of compounds found in the AD were benzophenones, obtained from natural products or by synthetic transformations [50]. This group displays many biological activities including antioxidant, anti-inflammatory [51], antitumor [52] and antimicrobial properties [53,54]. In addition, some studies have shown the activity of benzophenones against *H. pylori* [55,56], contributing to the healing effects of AD from *M. indica*. 

## Conclusions

In the present work, the aqueous decoction from leaves of *Mangifera indica* did not show acute toxicity but exhibited gastroprotective effects against several ulcerogenic agents. We also demonstrated that in this decoction of *M. indica* leaves the main bioactive molecules are xanthones (mangiferin) and benzophenone glycoside. 

## Experimental

### General

Structures of compounds were determined by combining the ^1^H-, ^13^C, and 2D-NMR techniques and by comparing their spectroscopic data with those reported in the literature [37]. NMR spectra in DMSO-*d*_6_ were obtained using a Varian INOVA 500 spectrometer, operating at 11.7 T. Chemical shifts are given as δ (ppm) using TMS as internal standard. Mass spectra were registered with a Termo Finnigan LCQ Deca ion-trap and Excalibur software. The sample was prepared in MeOH and analyzed in negative ion mode (capillary -4V, spray 5kV, lent offset 0V, capillary temperature 280º C, dry gas nitrogen at flow a rate of 50, scan 230-1800, injection time 50 ms, 3 microscans, collision energy 25%). The scan range was *m/z* 80-2.000. Analytical TLC was performed on Kieselgel 60 F254 (Merck) plates with 0.2 mm layer thickness. Spots were visualized by UV light or by spraying with anisaldheyde/H_2_SO_4_ solution after heading. Column chromatography (CC) was performed by gel permeation chromatography using a Sephadex LH-20 column (Pharmacia) and silicagel 60 (Merck). Compounds were purified using a semipreparative HPLC Knauer Chance system equipped with a Waters R401 refractive index detector, a Phenomenex Luna column (RP 18, 10 *μ*m, 250 × 10 mm) and a Rheodyne injector with a 100 *μ*L sample loop). All solvents for chromatographic separation were of analytical grade, from Synthlab. The chemicals and solutions used were all of analytical grade. All drugs and reagents were prepared immediately before use. The following substances and drugs were used: absolute ethanol, hydrochloric acid, acetic acid (Sinth, Brazil), cimetidine (Sigma, U.S.A), lansoprazole and piroxicam (Hexal, Brazil).

### Plant material, extraction and isolation

Leaves of *M. indica* were collected in Finca San José, Mazatenango, Suchitepéquez (Guatemala). Samples of the leaves were identified by the staff of Armando Cáceres and a voucher specimen is kept at the Herbarium of Farmaya Laboratory, Guatemala, under the number 810. For this study, *Mangifera indica* leaves were employed in decoction form obtained as follows: The powdered plant (300 g) was extracted with water (500 mL), boiled for 30 min. The mixture was filtered with Whatman number 1 filter paper, cooled and filtered a second time. The filtrate (AD) was lyophilized with a dry-drug yield of 13.05%. This extract was stored in a refrigerator at 10 ºC until used and dissolved in distilled water on the day of the experiment to prepare the stock solution and different dilutions for the purpose of pharmacological or chemical evaluation. 

The AD (2.0 g) was subjected to chromatography over gel permeation (10 x 2.5 cm i.d.) using MeOH as eluent. Fractions (5 mL) were collected and checked by TLC on silica-gel plates, mobile phase CHCl_3_/MeOH/*n*-PrOH/H_2_O (5:6:1:4, v/v, organic phase). The eluents were combined according to TLC control. Fraction 9 was sequentially purified by CC on silica gel (60 H, Merck) column (10 x 5 cm i.d.) and eluted using solvent mixtures composed of CHCl_3_/MeOH. The 80 % MeOH fraction afforded a yellow crystalline powder (15 mg) denominated Mi1. Fraction 5 was further purified using a HPLC-IR. Isocratic elution using MeOH/H_2_O (45:55, v/v) as mobile phase, at flow rate of 1.5 mL/min, yielded the benzophenone glycoside Mi2 (12 mg). 

### Quantitative determination of phenolic content in AD

The global polyphenol content of AD was determined by the Folin-Ciocalteu colorimetric method proposed by Singleton and Rossi [57] with some modifications. An aliquot of the extract (0.100 mg) was dissolved in MeOH (2 mL), and the extracts were diluted 10-fold with water. Folin–Ciocalteu reagent (0.25 mL, 1:1, v/v, Merck) was added to the diluted solutions (0.25 mL) then distilled water (2.0 mL) and a 200 g/L solution of Na_2_CO_3_ (0.25 mL) were added. After 30 min at room temperature, the absorbance was measured at 760 nm (Hach Dr-4000U spectrophotometer) with a blank sample (water plus reagents) in the reference cell. Quantification was expressed by reporting the absorbance in the calibration curve of gallic acid (Vetec®), used as phenol standard. All samples were analyzed in triplicate.

### Chemical fingerprint of AD

The chromatographic profile of AD was performed using a Varian, ProStar HPLC system equipped with an RP-18 column (250 mm × 4.6 mm i.d., 5 *μ*m, Phenomenex Luna). The mobile phase was water (A) and acetonitrile (B) starting with linear gradient elution of 21-30% of B in 30 min, 30-36% of B in 50 min and 36–100% in 60 min eluted at a flow-rate of 1.17 mL/min, and the effluent was monitored using a ProStar 330 photodiode-array ultraviolet detection (PDA) system. Spectral data from all peaks were registered in the range 200–400 nm. An aliquot (5.0 mg) of AD was dissolved in ultra-pure water and filtered with a 0.22 *μ*m membrane (Millipore) to an appropriate solution of 1.0 mg/mL. The injection volume for samples was 20 *μ*L onto the HPLC column. 

### Spectral data of compounds

*Mi1*: Yellow crystalline solid; mp 271 ºC; IR (KBr) ν_max_: 3.400 (OH), 1.645 (C=O) cm^-1^; UV (MeOH) λ_max_ 240, 259, 316, 365 nm. EI-MS m/z: 423 [M+H]^+^; H-NMR (DMSO-*d*_6_) δ: 7.36 (1H, s H-8), 6.84 (1H, s, H-5), 6.36 (1H, s, H4), 4.60 (1H, d, *J*=9.8 Hz, glc H-1), 3.19-4,04 (sugar prótons); ^13^C-NMR (DMSO-*d*_6_) δ: 177.1 (C-9), 162.3 (C-3), 160.2 (C-1), 154.8 (C-6, C4a), 149.5 (C-10a), 142.6 (C-7), 111.2 (C-8a), 107.7 (C-8), 107.3 (C-2), 102.4 (C-5), 101.1 (C-9a), 93.3 (C-4), 81.8 (C-5’), 79.3 (C-3’), 73.5 (C-1’), 71.1 (C-2’), 70.7 (C-4’), 62.2 (C-6’). 

*Mi2*: Brown amorphous solid; IR (KBr) ν_max_: 3.300 (OH), 1.615 (C=O) cm^-1^. UV (MeOH) λ_max_ 210, 294 nm. EI-MS m/z: 407 [M-H]^-^, 287 [M-H-120]^-^; ^1^H-NMR (DMSO-*d*_6_) δ: 7.55 (2H, d, *J*=8.0 Hz, H-2’, H-6’), 6.77 (2H, d, *J*=8.0 Hz, H-3’, H-5’), 5.95 (1H, s, H6), 4.59 (1H, d, *J*=10.0 Hz, glc H-1), 3.19-3.58 (sugar protons); ^13^C-NMR (DMSO-*d*_6_) δ: 194.6 (C-7), 161.3 (C-4’), 159.1 (C-4), 157.4 (C-3), 156.8 (C-2), 131.4 (C-2’, C-6’), 130.7 (C-1’), 114.6 (C-3’, C-5’), 106.9 (C-1), 103.6 (C-5), 94.9 (C-6), 81.0 (C-5’’), 78.3 (C-3’’), 74.6 (C-1’’), 71.9 (C-2’’), 69.6 (C-4’’), 60.5 (C-6’’). 

### Animals

Male Swiss albino mice (25-35 g) and male Wistar albino rats (150-250 g) from the Central Animal House of the UNESP were used. The animals were fed a certified Nuvilab^®^ (Nuvital) diet with free access to tap water under standard conditions of 12 h dark-12 h light, humidity (60 ± 1.0%) and temperature (21 ± 1%). Fasting was used prior to all assays because standard drugs and infusion were always administered orally (by gavage) or intraduodenally using a saline solution (10 mL/Kg) as the vehicle. Moreover, the animals were kept in cages with raised floors of wide mesh to prevent coprophagy. The number of animal was enough the statistical analysis and we reduced number of animals to respect the Ethical Animal Care. The animals were killed by CO_2_ gas (1 min with outflow 15 L/min) and all experiments following the recommendations of the Canadian Council on Animal Care [58] and all of the employed protocols were approved by UNESP Institutional Animal Care and Use Committee. 

### Acute toxicity

The acute toxicity studies of *M. indica* were performed in mice. In this assay, increasing doses of AD were orally administered to groups of ten animals for each dose after a 12 h fast. Animals receiving the vehicle (saline) served as control. The signs and symptoms associated with the AD administration (5 g/Kg, p.o.) were observed at 0, 30, 60, 120, 180 and 240 min after and then once a day for the next 14 days. At the end of the period the number of survivors was recorded. The acute toxicological effect was estimated by the method described by Souza Brito [59]. 

*Gastroprotective effect against different ulcerogenic agents*: HCl/ethanol-induced ulcer [60] - Mice were divided into groups (n=6-7) that had each fasted for 24 h prior to oral dosing with the vehicle (10 mL/Kg), lansoprazole (30 mg/Kg) and AD (250, 500 or 1,000 mg/Kg). Fifty minutes after the treatments, all animals received a 0.3 M HCl/60% EtOH solution (0.2 mL) orally. Animals were killed 1 h after the administration of HCl/EtOH solution. The stomachs were removed, opened along the greater curvature and fixed between two glass plates. Ulcerative lesions were calculated according to the methodology described by Szelenyi and Thiemer [61]. Indomethacin-induced gastric ulcers in mice according to Rainsford [62] - In this model, gastric lesions were induced with indomethacin (30 mg/Kg, s.c.) and administered to mice (n=7-8) after a 24 h fast. The AD (250, 500 or 1,000 mg/Kg), cimetidine (100 mg/Kg) or vehicle was administered orally 30 min before the induction of gastric ulcer. The animals were killed 4 h after treatment with the ulcerogenic agent. The stomachs were removed and ulcerative lesions were calculated as described previously. Absolute-Ethanol-induced damage [30] - After a total of 24 h fasting, five groups of rats (n=5) received an oral administration of AD (250, 500 or 1,000 mg/Kg), lansoprazole (30 mg/Kg) or vehicle (10 mL/Kg). One hour after treatment, all rats received one mL of 99.5% ethanol to induce gastric ulcer. The animals were killed one hour after treatment with the ulcerogenic agent and the stomachs removed to determine the lesion damage. Hypothermic Restraint Stress Ulcer [63]. After having fasted for 24 h, mice (n=7-8) received an oral administration of AD (250, 500 or 1,000 mg/Kg), cimetidine (100 mg/Kg) or vehicle (10 mL/Kg). One hour after treatment, mice were immobilized in a restraint cage at 4°C for 4 h to induce gastric ulcer. The animals were killed and the stomachs removed and opened along the greater curvature to determine the number of gastric lesions. The differences between the times of oral administration of AD are in accordance to animal species and metabolisms and all methods are in accordance to authors that standardized each one. 

### Statistical analysis

Results are expressed as mean ± S.E.M. Statistical significance was determined by one-way analysis of variance followed by Dunnett’s or Student’s test, with the level of significance at p < 0.05.
